# Structural Racism, Menthol Cigarettes, and Incident Lung Cancer Among Black Adults—Need to Revisit Evidence-Based Guidelines

**DOI:** 10.1001/jamanetworkopen.2025.18490

**Published:** 2025-07-01

**Authors:** Jennifer A. Campbell, Rebekah J. Walker, Leonard E. Egede

**Affiliations:** Division of Population Health, Department of Medicine, Jacobs School of Medicine and Biomedical Sciences, University at Buffalo, Buffalo, New York

Structural inequalities are characterized by the unequal distribution of power, resources, and access, creating and reinforcing disproportionate exposure to hazardous circumstances at the material, psychological, and behavioral levels for those in lower socioeconomic positions.^1^ When structural inequalities are primarily based on race and ethnicity, it is defined as structural racism.^2^ Structural racism perpetuates discrimination through mutually reinforcing systems and institutions, such as housing, employment opportunities, access to health care, and the built environment.^2^ Historic redlining and the resulting residential segregation by race and ethnicity is an expression of structural racism, and its impact on health outcomes is now measurable with current data sources.^3^

Another expression of structural racism is discriminatory media and marketing. Health-harming marketing strategies by the tobacco industry capitalized on residential segregation for decades through targeted media and marketing of menthol cigarettes.^4^ As evidence of the health-harming effects of tobacco emerged, tobacco companies ignored or tried to minimize this evidence and intentionally marketed menthol cigarette brands to the Black community, particularly low-income Black communities.^4^ The mutually reinforcing nature of structural racism was again shown in the 2009 controversial omission of menthol cigarettes from the Family Smoking Prevention and Tobacco Control Act, maintaining intentional exposure and distribution of menthol cigarettes. Today, Black US residents have the highest prevalence of menthol cigarette use among smokers of all racial and ethnic groups^5^ and are diagnosed with lung cancer at earlier rates, are less likely to receive timely treatment, and have higher mortality rates compared with individuals who belong to other racial ethnic groups.^6^ While extensive discourse exists on the history of targeted advertisement of menthol cigarettes to the Black community across marketing platforms, less has been done to quantify the role of menthol cigarette use as a pathway between structural racism as expressed by discriminatory media marketing and the incidence of lung cancer.

Xiao et al^7^ examine the association between residential segregation as a measure of structural racism and lung cancer risk among African American adults. This study specifically explored the mediating role of menthol cigarette use as well as environmental exposures, such as air pollution, and other sociodemographic factors. Using data from the Southern Community Cohort Study, representing 12 states from the southeastern United States, a cohort of 71 634 African American and White adults were included. Residential segregation was measured using the isolation index, and results show that African American adults were more segregated than their White counterparts in the cohort.^7^ Additionally, segregation was associated with higher incidence of lung cancer among African American participants.^7^ Menthol cigarette smoking, air pollution as measured by particulate matter with a diameter of 2.5 μm or less, secondhand smoke, and educational attainment all significantly mediated this association.^7^ Finally, using hypothetical scenarios to simulate the impact that reducing residential segregation may have on incident lung cancer, this study found that reducing residential segregation could reduce the 17-year risk of lung cancer risk for African American adults.^7^

This study represents an important step in quantifying the influence of structural racism on disease onset through hazardous exposure in the built environment and highlights the enduring effects of structural racism on health outcomes for Black US residents. Given the analysis only captures segregation across the southeastern region of the United States, these findings may underestimate the magnitude of this association. Results highlight the importance of work to identify additional areas for intervention. While the mediation analysis showed that 47% of the association between residential segregation and lung cancer risk was accounted for by menthol cigarette smoking, air pollution, secondhand smoke, and educational attainment, 53% of the variance was unexplained, suggesting other pathways may be at play. Additionally, this study suggests that lung cancer risk can be reduced in African American communities by addressing the lingering effects of residential segregation, including through interventions that target key pathways of menthol smoking, secondhand smoke, air pollution, and educational attainment for African American adults.

Interventions that aim to mitigate the impact of structural racism must be intentional in incorporating key mechanisms that may have historic origin yet continue to have lingering effects on health outcomes. Specifically, there is need to address the detrimental effects of historic discriminatory media and marketing and their lingering effects on lung cancer outcomes for Black adults. As structural racism is mutually reinforcing, not operating through one singular mechanism but through multiple sectors, policy-based solutions must also include multifaceted approaches that encompass multiple mechanisms.^3^ Prior work has highlighted the importance of policies that address (1) economic empowerment through tax incentives, employment programs, educational opportunities, and home ownership coordination; (2) improving the built environment through greenspace development; creation of sidewalks, bike paths, and walking paths; and investment in public transportation; (3) food access programs that expand supermarket placement and incentivize healthy eating choices; (4) expansion of health care access and affordability; and (5) investments in housing development.^3^ However, media and marketing are often not the focus of policy initiatives or policy interventions within these sectors. Since media and marketing are key channels that drive societal values and social identities, future research should examine how they serve as mechanisms and pathways for the relationship between structural inequalities and racism and health outcomes across populations.

Similarly, guidelines for lung cancer screening need to be revisited with recent data on the impact of structural inequalities on lung cancer outcomes in mind. Current US Preventive Services Task Force (USPSTF) recommendations state that “Adults aged 50 to 80 years who have a 20 pack-year smoking history and currently smoke or have quit within the past 15 years are considered meeting eligibility for screening.”^8^ While the USPSTF notes other risk factors, such as Black men having a higher incidence, current guidelines do not include different screening recommendations for those at higher risk. Given the history of targeted marketing by the tobacco industry to specific populations, most notably the Black community, elaborate risk prediction models^8^ that largely focus on individual-level factors may not be the solution for reducing incidence and mortality rates among communities that are at disproportionately increased risk of developing lung cancer. Rather, as the evidence from Xiao et al^7^ shows, populations disproportionately exposed to targeted marketing for menthol cigarettes, secondhand smoke, or higher levels of air pollution within their community may benefit from earlier screening or lower cutoffs in terms of pack-years for eligibility.

In summary, to effectively address the accumulating body of evidence on the effects of structural inequalities and racism on health outcomes, new strategies are needed. These strategies need to go beyond token interventions or interventions directed at the individual to more population health–focused strategies that account for mutually reinforcing effects at the neighborhood and community levels. This will require cross-sector collaborations, incorporation of emerging evidence on the impact of structural determinants on individual health outcomes into clinical guidelines, and a different form of advocacy that is based on broad coalitions of patient groups, community groups, health systems, and relevant professional organizations, with the singular focus of improving health outcomes for all US residents. For lung cancer disparities, we have enough evidence, we need to reevaluate current screening and treatment guidelines, and the time to act is now.
